# First Report on Rigid Plate Fixation for Enhanced Sternal Closure in Minimally Invasive Cardiac Surgery: Safety and Outcomes

**DOI:** 10.3390/bioengineering11121280

**Published:** 2024-12-16

**Authors:** Jules Miazza, Benedikt Reuthebuch, Florian Bruehlmeier, Ulisse Camponovo, Rory Maguire, Luca Koechlin, Ion Vasiloi, Brigitta Gahl, Luise Vöhringer, Oliver Reuthebuch, Friedrich Eckstein, David Santer

**Affiliations:** 1 Department of Cardiac Surgery, University Hospital of Basel, 4031 Basel, Switzerland; jules.miazza@usb.ch (J.M.); benedikt.reuthebuch@usb.ch (B.R.); floriansamuel.bruehlmeier@usb.ch (F.B.); ulisse.camponovo@stud.unibas.ch (U.C.); roryjames.maguire@usb.ch (R.M.); luca.koechlin@usb.ch (L.K.); ion.vasiloi@usb.ch (I.V.); brigitta.gahl@usb.ch (B.G.); luise.voehringer@usb.ch (L.V.); oliver.reuthebuch@usb.ch (O.R.); friedrich.eckstein@hirslanden.ch (F.E.); 2 Medical Faculty, University Basel, 4056 Basel, Switzerland; 3 Center for Biomedical Research and Translational Surgery, Medical University of Vienna, 1090 Vienna, Austria

**Keywords:** cardiac surgery, rigid plate fixation, enhanced sternal closure, aortic valve replacement, ministernotomy, partial upper hemisternotomy, sternal wound infection

## Abstract

Introduction: This study reports of the use of a rigid-plate fixation (RPF) system designed for sternal closure after minimally invasive cardiac surgery (MICS). Methods: This retrospective analysis included all patients undergoing MICS with RPF (Zimmer Biomet, Jacksonville, FL, USA) at our institution. We analyzed in-hospital complications, as well as sternal complications and sternal pain at discharge and at follow-up 7 to 14 months after surgery. Results: Between June and December 2023, 12 patients underwent RPF during MICS, of which 9 patients were included in the study. The median (IQR) age was 64 years (63 to 71) and two patients (22%) were female. All patients underwent aortic valve replacement, with two patients (22%) undergoing concomitant aortic surgery. RPF was successfully performed in all patients. ICU and in-hospital stay were 1 day (1 to 1) and 9 days (7 to 13), respectively. Patients were first mobilized in the standing position on postoperative day 2 (2 to 2). Four patients (44%) required opiates on the general ward. In-hospital mortality was 0%. At discharge, rates of sternal pain, sternal instability or infection were 0%. After a follow-up time of 343.6 days (217 to 433), median pain intensity using the Visual Analog Scale was 0 (0 to 2). Forty-four percent (*n* = 4) of patients reported pain at rest. No sternal complications (sternal dehiscence, sternal mal-union, sternal instability, superficial wound infections and deep sternal wound infections) were reported. Conclusions: In the evolving landscape of cardiac therapies with incentives to reduce surgical burden, RPF showed safety and feasibility. It might become an important tool for sternal closure in minimally invasive cardiac surgery.

## 1. Introduction

Full median sternotomy followed by steel wiring has been the standard approach in cardiac surgery for the last decades. In recent years, the advent of interventional cardiac therapies and the increasing use of transcatheter valve replacement strategies [1] has created an appeal for less invasive surgical strategies.

Minimally invasive cardiac surgery (MICS), performed via a partial upper hemisternotomy or a right anterior thoracotomy, has been proposed as an alternative to median sternotomy for the treatment of aortic valve diseases [2,3]. However, it remains unclear if postoperative sternal complications, such as sternal pain, sternal instability or sternal infection, can be reduced by MICS [4,5,6] Previous studies showed that the risk of sternal complications is comparable between MICS and full sternotomy [7,8]. Therefore, with the increasing number of MICS procedures, enhanced sternal closure solutions must be discussed.

Rigid plate fixation (RPF) has been proposed as an alternative to conventional steel wiring [9,10]. In patients undergoing full-sternotomy, RPF has been associated with improved bone healing and a reduction in postoperative pain [11,12]. Furthermore, RPF has showed reduced sternal bone movement at 12 weeks after full sternotomy when assessed with ultrasound [13]. While results of RPF after full sternotomy are promising, data on RPF after MICS are scarce.

**Figure 1:**
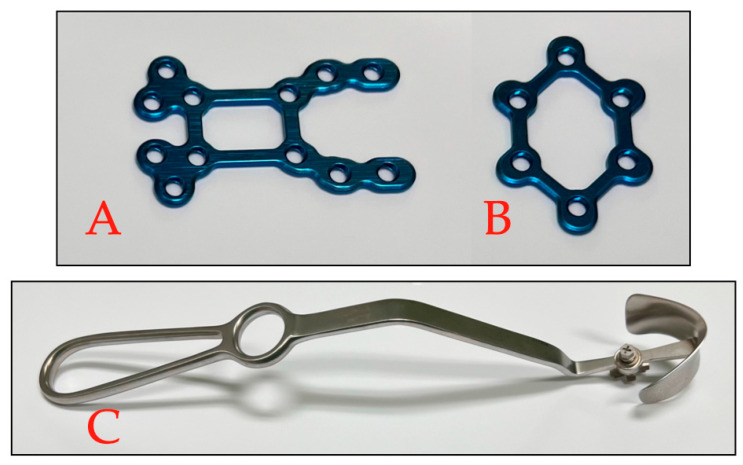
SternaLock Blu MICS system. (**A**) MICS osteosynthesis plate; (**B**) hexagonal plate; (**C**) skin retraction tool.

Aiming to further improve outcome after MICS, RPF was introduced at our institution in 2023. The systems used are the SternaLock Blu system (Zimmer Biomet, Jacksonville, FL, USA), and the SternaLock Blu MICS system (Zimmer Biomet, Jacksonville, FL, USA). Both systems consist of titanium osteosynthesis plates designed for sternal closure. Additionally, the SternaLock Blu MICS system is equipped with dedicated hexagonal (6-Loch-Hexagon-Platte, SP-2890) and MICS (modifizierte 12-Loch-JL-Platte, SP-3215) plates (Figure 1A,B) and a skin retraction tool to help sternal approximation and reduce skin incision (Figure 1C).

This study aims to assess the safety and feasibility of RPF in patients undergoing MICS. We hypothesized that patients treated with RPF after MICS would experience few complications and low levels of postoperative pain. To the best of our knowledge, we provide the first report including follow-up data on the use of an RPF system in patients treated with MICS.

## 2. Materials and Methods

### 2.1. Data Collection and Definitions

This is a retrospective, single-center database analysis performed at the Department of Cardiac Surgery, University Hospital of Basel, Switzerland. Since 2023, twelve patients underwent MICS with subsequent RPF. We included patient with diagnosis of severe aortic stenosis according to the 2021 ESC/EACTS Guidelines for the management of valvular heart disease [14] and suitable anatomy for MICS. Patients requiring concomitant procedures necessitating full sternotomy (i.e., aortocoronary bypass) were excluded. Patients’ informed consent for the operation was obtained as per institutional standard using a patient consent form after detailed patient information and answering technical questions. RPF was routinely used after MICS at our institution at the time of the study. The final choice of the closure system was left to the operating surgeon.

Patient data were collected using an institutional, prospectively maintained quality management database (Dendrite Clinical Systems, V1.7, Reading, UK). This database is regularly checked for consistency and completeness. After informed consent was obtained from the patients, follow-up data were collected from patients´ medical records, as well as from telephone interviews using a specific case report form. Follow-up data were then entered in a dedicated, good clinical practice (GCP) conform, RedCap database. Follow-up was performed 7 to 14 months after the index surgery using a standardized case report form.

The Center for Disease Control and Prevention’s definition of superficial and deep wound infections was used for the definition of sternal infectious complications [15]. Sternal pain was characterized as any discomfort localized at the surgical site, excluding symptoms indicative of myocardial ischemia. Sternal mal-union referred to a partial or complete lack of fusion between the sternal halves, as per existing literature [16]. Sternal dehiscence was described as the separation of sternal halves accompanied by instability, pain, instability, or necessitating subsequent surgical intervention [17].

Data are expressed as mean (standard deviation), median (quartiles), and frequency with percentage, based on the nature and distribution of the data. Perioperative and follow-up outcomes were defined according to the updated Valve Academic Research Consortium 3 (VARC-3) [18].

### 2.2. Surgical Technique

#### 2.2.1. Minimally Invasive Cardiac Surgery

**Figure 2:**
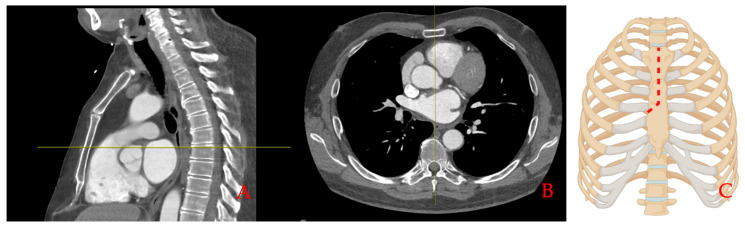
Surgical access in MICS. Surgical planning for MICS: (**A**,**B**) envisioned sternum section plane using multiplane CT scan; (**C**) J-shaped ministernotomy (created with BioRender.com).

At our institution, MICS is routinely performed by a J-shaped upper hemisternotomy. The surgical strategy for MICS has been described previously [19]. Briefly, surgical planning is based on preoperative imaging and patient anatomy (Figure 2A,B). The surgical setting is similar to full sternotomy with the addition of external defibrillation patches. A 5 to 7 cm skin incision starting under the sternal notch is performed and the subcutaneous tissue is dissected down to the sternum. The sternum is dissected using either an oscillating or a regular saw down to the level of the third or fourth intercostal space. Then, an oblique incision is made, and the right sternal half is separated using the saw (Figure 2C). Following the opening of the pericardium, cardiopulmonary bypass is established either centrally or using femoral venous canulation depending on the surgical situs. Subsequently, aortic valve replacement and/or aortic surgery is performed in standard fashion.

#### 2.2.2. Sternal Closure Using Rigid Plate Fixation

**Figure 3:**
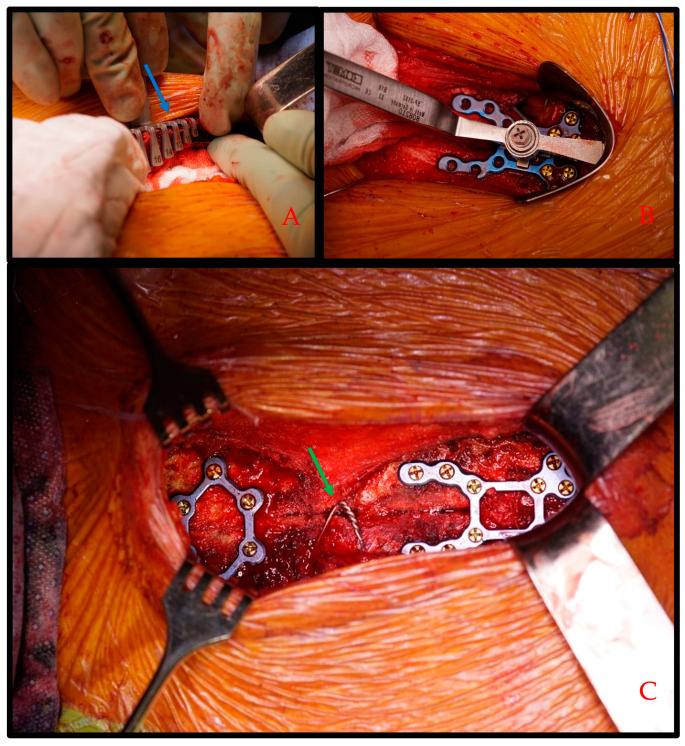
Sternal closure using rigid plate fixation. Rigid plate fixation after minimally invasive cardiac surgery. In all intraoperative images, the cranial is to the left and the caudal is to the right. (**A**) Measurement of screw length using a specific designed tool (blue arrow) prior to rigid plate fixation to avoid loose screws or perforation or dorsal bone cortex. (**B**) Fixation of the caudal plate with a holding tool and pre-measured screws. The MICS plate is mounted on the skin retractor to help with plate positioning. (**C**) Completed rigid plate fixation. A conventional sternal wire is used to optimize approximation (green arrow).

After completion of MICS procedure, hemostasis is performed and the pericardium closed followed by sternal closure. In this cohort, sternal closure was performed with RPF using either the SternaLock Blu [12] or SternaLock Blu MICS systems. RPF systems are designed similarly to osteosynthesis material used in orthopedic surgery. Consequently, the instruments and RPF plates are prepared and sterilized according to our institutional protocols and the manufacturer’s instructions for use [20]. Sternal closure with RPF is performed as follows. First, the sternal bone thickness is measured using a designed sternal thickness measurement tool (Figure 3A, blue arrow) at the level of the manubrium, at the third intercostal space and at the inflexion point of the J-shaped ministernotomy. These measurements allow the definition of screw length for sternal plating, thus preventing loose screws and the usage of too long screws but simultaneously ensuring the anchoring of the dorsal bone cortex [21]. In our experience, the use of a conventional steel around the manubrium helps the approximation and fixation of the sternal parts. The caudal plate is then fixed on the distal sternal half, followed by the approximation and then fixation of the cranial half of the osteosynthesis plate (Figure 1B). It is important to note that the fixation of the second sternum half should be performed under traction to allow for better approximation. Next, the remaining plate is placed at the manubrium and the third intercostal place, following the same technique. (Figure 1C). As per our institutional standard, we rinse the wound with aqua and apply vancomycin between the sternum halves prior to sternal closure to reduce postoperative sternal infections [22]. We then perform skin closure in a standard fashion. It is of note that, in case of urgent re-sternotomy, RPF osteosynthesis plates can be cut open using a standard straight or angulated tip wire cutter.

## 3. Results

### 3.1. Baseline Characteristics

From June to December 2023, twelve patients underwent MICS followed by RPF at our institution, of which nine were eligible for this study. The median age (IQR) was 64 (63 to 71) and 22% (*n* = 2) were female. The median body mass index was 26.3 (22 to 30.6). The left ventricular ejection fraction (LVEF) was preserved [64 (59 to 65)]. Further patient characteristics are depicted in Table 1.

### 3.2. Operative Data

In all patients, aortic valve replacement was performed via MICS. The indication for aortic valve replacement was severe aortic stenosis in 75% (*n* = 9) and severe aortic valve insufficiency in 25% (*n* = 3). In one case (8%), the concomitant replacement of the ascending aorta and hemiarch was performed due to dilatation. The median operative time, cardiopulmonary bypass time and aortic cross clamping time were 209 (197.5 to 223), 112.5 (104.5 to 116.5) and 83 (59.3 to 86.3), respectively. Intraoperative data is depicted in Table 2.

### 3.3. Postoperative Data

We observed no operative mortality. The median length of intensive care unit stay was 1 day (1 to 1), and the median length of hospital stay 8.5 days (7.8 to 10.8). There was one case of postoperative stroke (8%) and two patients (17%) developed atrial fibrillation. Analgesia using opiate was required in 42% (*n* = 5) once transferred to the cardiac surgery department. There were no cases of sternal infection, pain or instability at discharge. The values of C-reactive protein and white blood cells count peaked on postoperative day 2 and 3, respectively. Postoperative data is depicted in Table 3 and postoperative values of C-reactive protein and white blood cells counts in Table 4.

### 3.4. Follow-Up Data

**Figure 4:**
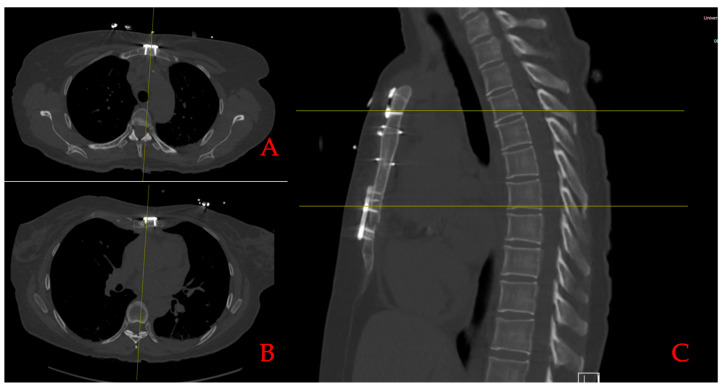
Postoperative CT scan after rigid plate fixation. Postoperative CT scan showing ossification and correct plate position of the rigid plate fixation system at the level of (**A**,**C**) the manubrium and (**B**,**C**) the corpus sterni.

Follow-up was obtained from all included patients (*n* = 9). We report no superficial or deep sternal infections. Sternal stability was achieved in all patients. In three patients (33%) with available CT scans, ossification was satisfactory (Figure 4). Follow-up data is depicted in Table 5.

## 4. Discussion

Data of rigid plate fixation following minimally invasive cardiac surgery are scarce. In a case-series from 2014, Russo et al. [23] reported on RPF after MICS using the ministernotomy approach. They showed promising results with no mortality, stroke and, importantly, no sternal wound infections. Except for this study, data on RPF after MICS remain almost non-existent.

In this retrospective, single-center study we report the first data on the use of RPF following MICS via J-shaped ministernotomy including follow-up. Between June and December 2023, 12 patients underwent MICS followed by RPF at our institution. Nine patients consented to take part in this study. RPF was successful in all cases with no complications linked to the closure system. Furthermore, the incidence of secondary safety outcomes such as acute kidney injury, stroke, atrial fibrillation and systemic infections was unremarkable and were clinically unlikely to be related to the RPF system. The postoperative C-reactive protein and white blood cell values peaked on day two and three, as expected after cardiac surgery [24]. There was no sternal complication at discharge. No sternal instability, superficial or deep wound infections were reported at follow-up. Three patients (33%) reported pain at rest with a median VAS score of 0 (0 to 2), while no patient required analgesics. One patient reported sternal pain after coughing.

### 4.1. Evolution of Surgical Access and Sternal Closure Techniques

Full median sternotomy, followed by closure with steel wires has been the access of choice for cardiac surgeons for decades. While this technique yields satisfactory results in most cases, sternal complications still represent a burden for cardiac surgery patients, including sternal pain, sternal infections or sternum dehiscence or non-union.

In recent years, MICS has been continuously refined to mitigate surgical trauma with results showing reduced length of ICU stay, blood loss and total length of hospital stay in patients treated with MICS vs ministernotomy [6]. However, a Cochrane Database Systematic Review performed in 2017 by Kirmani et al. [25], showed no difference in postoperative score pain or sternal infections, while confirming a slight reduction in length of ICU stay. This contrasted data on the outcome after MICS regarding sternal complications warrants further analyses and encourages the search for further optimization strategies regarding postoperative pain, sternal infections and sternal instability. To palliate such complications, the 2019 Guidelines for Perioperative Care in Cardiac Surgery: Enhanced Recovery after Surgery Society Recommendations recommend enhanced sternal fixation in selected patient groups [26].

### 4.2. Post-Sternotomy Pain

Chronic post-sternotomy pain is a complication affecting 28 to 39% of patients after cardiac surgery [27,28]. Rigid plate fixation has been proposed as an alternative to metal wires for sternal closure after full sternotomy. This approach seems to positively affect postoperative pain. In a randomized controlled multicenter trial published in 2012, Raman et al. showed that RPF reduced sternal pain at 3 weeks when compared to sternal wiring. In 2017, Allen et al. reported improved patient reported outcome measures regarding the symptoms of pain in patients treated with RPF vs sternal wiring [29]. In our cohort, 33% (*n* = 3) of patients reported pain at rest. Interestingly, fewer patients reported pain after coughing (11%, *n* = 1) than at rest, which is in contrast with the available literature, typically showing more pain after coughing [29]. This might be owing to the fact that the available literature reports on patients treated via full median sternotomy and not MICS. Furthermore, in our cohort, the mean pain score using the VAS scale was 0 (0 to 2). According to the literature, on a scale of 100 mm, 4 to 44 mm is considered mild pain. Consequently, postoperative pain in our cohort is situated on the lower half of the mild pain category. Our findings are consistent with existing literature, with confirmed improved sternal stability and low pain scores after RPF in full sternotomy [11]. These findings are encouraging in the quest to reduce sternal pain after cardiac surgery.

### 4.3. Sternal Wound Infections

Previous studies have shown deep sternal wound infection to be a catastrophic complication with an incidence 1 to 5% and a mortality rate up to 47% [30,31,32]. In 2006, Raman et al. studied the use of RPF vs. full median sternotomy in patient at high risk of mediastinitis [33]. They reported a decreased rate of mediastinitis (0% vs 13%, *p* < 0.05) after a follow-up which ranged from 4 to 200 weeks. In their randomized multicenter trial in 2017, Allen et al. showed a trend towards reduced sternal infections (0% [0/116] vs. 4.2% [5/120]; *p* = 0.06) [34]. In our cohort, we reported no superficial or deep sternal wound complications, enlightening the potential role of a MICS and RPF combination to reduce rates of infectious sternal complications. This low level of infections could be the result of a greater sternal stability offered by RPF, as described in previous studies [34]. Indeed, sternal instability can be a consequence of sternal wound infections but also a precursor. Consequently, reducing sternal instability might help reduce the rate of sternal infections.

### 4.4. Limitations

This study has the following limitations. First, the retrospective, descriptive design does not allow for a comparison of RPF and traditional sternal wiring, or a comparison of full sternotomy vs MICS. As RPF is not the only closure system at our institution, and the decision to perform RPF was left to each surgeon, there is relevant risk of selection bias in our patient cohort. Furthermore, even though clinical follow-up was obtained, imaging follow-up was not routinely performed. In future studies, sternal bone assessment using ultrasound should be considered, as it has been performed to assess for sternal bone movement in the past [13]. Finally, even though no sternal complications leading to reoperation was recorded at follow-up, the general rate of sternal complications needing redo surgery is low at our institution (2.3%). In addition, the small sample size does not allow to draw definite conclusions on the infection rate and potential correlation between RPF and outcomes such as postoperative stroke, atrial fibrillation or systemic infections. Consequently, a more strongly powered analysis is needed to capture such complications after RPF.

## 5. Conclusions

In this retrospective, single-center analysis, we describe that MICS followed by RPF is safe and feasible. To the best of our knowledge, this is the first report including follow-up on patients undergoing MICS via J-shaped ministernotomy followed by RPF. In the future, RPF might be considered to reduce the postoperative burden of patients after MICS. Further prospective, comparative analysis are required to determine the role that RPF might play after minimally invasive cardiac surgery and should focus on a comprehensive comparison between RPF and conventional sternal wiring after MICS.

## Figures and Tables

**Table 1:** Baseline characteristics.

Baseline Characteristics	Total (N = 9)
Age, years	64 (63 to 71)
Female	2 (22%)
Diabetes	1 (11%)
BMI, kg/m^2^	26.3 (22 to 30.6)
Coronary artery disease	2 (22%)
Preoperative atrial fibrillation	1 (11%)
Periphery artery disease	0 (0%)
Previous cerebrovascular event	1 (11%)
Pulmonary disease including COPD	0 (0%)
Previous myocardial infarction	0 (0%)
Arterial hypertension	7 (78%)
Dyslipidemia	7 (78%)
Smoking history	
Never smoked	3 (33%)
Cessation	4 (44%)
Active	1 (11%)
Unknown	1 (11%)
Dyspnea Grade	
NYHA I	3 (33%)
NYHA II	3 (33%)
NYHA III	3 (33%)
NYHA IV	0 (0%)
Left ventricular ejection fraction, %	64 (59 to 65)
Preoperative mean gradient of stenosis, mmHg	51 (48 to 58.5)

Data are presented as mean and standard deviation (SD), median and interquartile range (IQR) or number and %. BMI body mass index, COPD chronic obstructive pulmonary disease, NYHA New York Heart Association.

**Table 2:** Operative data.

Intraoperative Data	Total (N = 9)
Aortic valve procedure	9 (100%)
Native aortic valve stenosis	7 (78%)
Native aortic valve regurgitation	2 (22%)
Type of prosthesis	
Biological	8 (89%)
Mechanical	1 (11%)
Size of valve prosthesis, mm	25 (24 to 27)
Prosthesis manufacturer	
Edwards© Perimount Magna ease	4 (44%)
Edwards© Inspiris Resilia	4 (44%)
Medtronic© Open Pivot AP 360	1 (11%)
Aortic procedure	2 (11%)
Emergency procedure	0 (0%)
Reoperation	0 (0%)
EuroSCORE II	0.77 (0.68 to 2.21)
Duration of surgery, min	208 (202 to 223)
Cardiopulmonary bypass time, min	112 (105 to 115)
Aortic cross clamp time, min	83 (60 to 84)
Successful RPF	9 (100%)
RPF with Sternalock Blu	4 (44%)
RPF with Sternalock Blu MICS	5 (56%)

RPF Rigid plate fixation. Data are presented as mean and standard deviation (SD), median and interquartile range (IQR) or number and %.

**Table 3:** Postoperative data.

Postoperative Data	Total (N = 9)
In-hospital mortality	0 (0%)
Length of ICU stay, days	1 (1 to 1)
Length of hospital stay, days	9 (7 to 13)
Rethoracotomy for bleeding	1 (11%)
Postoperative myocardial infarction	0 (0%)
Postoperative stroke	1 (11%)
Time to first mobilization, d	2 (2 to 2)
Atrial fibrillation at discharge	1 (11%)
Renal complication	0 (0%)
Infection	0 (0%)
Opiate need post-ICU	4 (44%)
Sternal pain at discharge	0 (0%)
Sternal instability at discharge	0 (0%)
Sternal infection at discharge	0 (0%)

Data are presented as mean and standard deviation (SD), median and interquartile range (IQR) or number and %.

**Table 4:** Postoperative values of C-reactive protein and white blood cells.

	Total (N = 9)
**Postoperative CRP (mg/L)**	
POD 1	57.8 (26.7)
POD 2	174.8 (50.8)
POD 3	158.6 (41.9)
POD 4	130.6 (21.3)
POD 5	75.7 (28.1)
Discharge	43.8 (17.8)
**Postoperative WBC count (G/L)**	
POD 1	11.3 (1.9)
POD 2	9.7 (1.2)
POD 3	9.8 (1.4)
POD 4	7.3 (1.5)
POD 5	7.1 (1.9)
Discharge	9.0 (1.4)

Data are presented as mean and standard deviation (SD). CRP: C-reactive protein, POD: postoperative day, WBC: white blood cells.

**Table 5:** Follow-up data.

Follow-Up Data	Total (N = 9)
Follow-up time, days	343.6 (217 to 433)
Superficial wound infections	0% (*n* = 0)
Deep wound infections	0% (*n* = 0)
Sternal instability	0% (*n* = 0)
Sternal pain at rest	44% (*n* = 4)
Pain intensity using Visual Analog Scale	0 (0 to 2)
Sternal pain after coughing	11% (*n* = 1)
Use of analgesics	0% (*n* = 0)
Revision linked to RPF	0% (*n* = 0)
CT scan available	33% (*n* = 3)
Sternal dehiscence	0% (*n* = 0)
Sternal mal-union	0% (*n* = 0)

This table depicts the study outcomes after a follow-up period of 7 to 14 months, obtained during telephone interviews. Data are presented as mean and standard deviation (SD), median and interquartile range (IQR) or number and %.

## Data Availability

The data presented in this study are available on reasonable request from the corresponding author.
